# Monitoring the susceptibility of *Bemisia tabaci* Middle East-Asia Minor 1 (Hemiptera: Aleyrodidae) to afidopyropen, cyantraniliprole, dinotefuran, and flupyradifurone in south Florida vegetable fields

**DOI:** 10.1093/jee/toae104

**Published:** 2024-05-15

**Authors:** Marcelo Dimase, Bruno Rossitto De Marchi, Felipe Barreto da Silva, Sriyanka Lahiri, Julien Beuzelin, Sam Hutton, Hugh Adam Smith

**Affiliations:** Department of Entomology and Nematology, Gulf Coast Research and Education Center, University of Florida, Wimauma, FL 33598, USA; Department of Entomology and Nematology, Gulf Coast Research and Education Center, University of Florida, Wimauma, FL 33598, USA; Department of Entomology and Nematology, Gulf Coast Research and Education Center, University of Florida, Wimauma, FL 33598, USA; Department of Entomology and Nematology, Gulf Coast Research and Education Center, University of Florida, Wimauma, FL 33598, USA; Department of Entomology and Nematology, Everglades Research and Education Center, University of Florida, Belle Glade, FL 33430, USA; Department of Horticultural Sciences, Gulf Coast Research and Education Center, University of Florida, Wimauma, FL 33598, USA; Department of Entomology and Nematology, Gulf Coast Research and Education Center, University of Florida, Wimauma, FL 33598, USA

**Keywords:** sweetpotato whitefly, insecticide resistance, neonicotinoid insecticide, diamide insecticide, *Bemisia tabaci* MEAM1

## Abstract

*Bemisia tabaci* Middle East-Asia Minor 1 (MEAM1) is a significant pest that damages a wide range of high-value vegetable crops in south Florida. This pest has demonstrated the ability to develop resistance to various insecticide groups worldwide. Monitoring the resistance levels of MEAM1 populations and maintaining baseline susceptibility data are crucial for the long-term effectiveness of insecticide management strategies. We conducted serial dilution bioassays on 15 field populations of MEAM1 collected in south Florida to assess their resistance to 4 key insecticides: afidopyropen, cyantraniliprole, dinotefuran, and flupyradifurone. To quantify resistance levels, resistance ratios (RR) were generated by comparing the LC_50_ values of field populations to those of a known susceptible MEAM1 colony reared in the laboratory. Our findings reveal that all field-collected populations were susceptible to dinotefuran (RR 1–8) and flupyradifurone (RR 2–8). While over 80% of the populations tested were susceptible to afidopyropen (RR 1–9), 2 populations exhibited low (RR 38) and moderate resistance (RR 51), respectively. In contrast, most of the populations (57%) showed low to moderate resistance to cyantraniliprole (RR 21–78), and the remaining populations were susceptible (RR 3–10). The 2 populations with resistance to afidopyropen also exhibited moderate resistance to cyantraniliprole. Further research in this direction can aid in refining insecticide resistance management programs in Florida and other regions where *B. tabaci* MEAM1 is a major pest. Exploring the implications of these findings will be essential for insecticide use and integrated pest management strategies in south Florida.

## Introduction

*Bemisia tabaci* (Gennadius) (Hemiptera: Aleyrodidae), also known as the sweetpotato whitefly, remains a predominant threat to a wide variety of crops, including tomatoes (*Solanum lycopersicum* L.) (Solanaceae) and other vegetables (Li et al. 2021). Previous research has shown that *B. tabaci* is not just a single species, but a complex of multiple cryptic species identified by mitochondrial cytochrome c oxidase subunit 1 (COI) DNA sequence (Perring 2001, De Barro et al. 2011). These cryptic species, although seemingly identical in morphology, vary genetically and can manifest differences in behavior, host choices, associated plant disorders, virus transmission capacities, and susceptibility to insecticides (Bedford et al. 1994, Perring 2001, De Barro et al. 2011, Polston et al. 2014, Rossitto De Marchi et al. 2017, Libardi Miraldo et al. 2021). The *B. tabaci* species complex comprises the globally dispersed Middle East-Asia Minor 1 (MEAM1) species, previously referred to as the B biotype, and the Mediterranean (MED) species, formerly known as the Q biotype. Recent research highlights the role of *B. tabaci* MEAM1 as a major pest of tomato crops in Florida (Rossitto De Marchi and Smith 2021).

The ability of *B. tabaci* to transmit the tomato yellow leaf curl virus (TYLCV) can have severe implications for yield and the quality of the fruit (Picó et al. 1996), and the direct damage caused by MEAM1 nymphs feeding on plant phloem can lead to plant disorders such as squash silverleafing (Hoelmer et al. 1991) and tomato irregular ripening (Schuster 2001). The agricultural communities in Florida have predominantly relied on synthetic insecticides to combat *B. tabaci* MEAM1 in open fields (Adkins et al. 2011, Smith and Giurcanu 2013, Smith et al. 2016, Rossitto De Marchi and Smith 2021, Rossitto De Marchi et al. 2021). However, exposing sequential generations of a pest population to the same modes of action contributes to the development of insecticide resistance (Georghiou 1983). Insecticide resistance arises when consistent use of an insecticide, as directed on the product label, ceases to effectively control a pest population (IRAC 2022). This control failure among *B. tabaci* populations is often attributed to resistance mechanisms found in several pest species. These mechanisms include mutations that confer resistance at the insecticide target site and/or an increase in detoxification enzyme levels (Lahm et al. 2019).

Among the more recent rotation strategies deployed to control *B. tabaci* in Florida, the systemic insecticides dinotefuran, flupyradifurone, and cyantraniliprole play a crucial role (Rossitto De Marchi et al. 2021). Dinotefuran is a nicotinic acetylcholine receptor (nAChR) agonist that belongs to Insecticide Resistance Action Committee (IRAC) mode of action (MoA) Group 4A. The nAChR is a protein that mediates the fast synaptic transmission, affecting whiteflies by disrupting their nervous system (Wakita 2008). Classified as a butenolide insecticide, flupyradifurone (IRAC MoA 4D) functions as an agonist to the nAChR of piercing-sucking insects such as aphids and whiteflies, mirroring the function of dinotefuran (Nauen et al. 2014, Jeschke et al. 2015). Cyantraniliprole (IRAC MoA 28) acts as a ryanodine receptor modulator, impacting whiteflies by targeting their calcium signaling pathways (Lahm et al. 2019). Furthermore, afidopyropen (IRAC MoA 9D) provides an alternative mode of action by targeting whiteflies’ chordotonal organs, disrupting their sensory functions (Horikoshi et al. 2022). Collectively, these diverse mode of action groups constitute a repertoire of active ingredients that are fundamental to the chemical management of whiteflies. Dinotefuran and flupyradifurone are commonly applied during the initial growth stages of tomato crops, particularly when it is crucial to protect them against virus transmission (Smith et al. 2018). Cyantraniliprole is best applied in the later stages of crop growth to provide additional control against leafminers and caterpillars (Rossitto De Marchi et al. 2021). Application methods may vary, including soil drench, drip irrigation systems, or foliar application, depending on the specific formulation and the crop’s developmental stage. A previous study indicated reduced efficacy of some insecticides, including cyantraniliprole, in managing *B. tabaci* populations across Florida (Rossitto De Marchi et al. 2021). Additionally, resistance monitoring has not been conducted for afidopyropen in Florida since it was first made available in 2018. Therefore, monitoring the susceptibility of whitefly populations across south Florida to key insecticides is critical, and serial dilution bioassays have been a vital methodology to determine resistance patterns among whitefly populations (Castle et al. 2010, Smith et al. 2016, Hopkinson and Pumpa 2019).

Bioassays generate dose–response curves to establish baseline susceptibility data. Comparing the dose–response of field-collected populations to a reference susceptible laboratory colony facilitates the understanding of prevailing resistance levels. Our investigation aimed to monitor *B. tabaci* MEAM1 resistance to dinotefuran, flupyradifurone, afidopyropen, and cyantraniliprole across south Florida. In such landscapes, whitefly populations may experience different selection pressures and develop varying levels of insecticide tolerance. Thus, we hypothesized that *B. tabaci* MEAM1 populations across south Florida would exhibit a diverse spectrum of responses to these insecticides. The primary goal of this study was to evaluate the extent and geographical distribution of resistance to key insecticides for managing *B. tabaci* MEAM1 in south Florida. Such information is essential to refine insecticide resistance management (IRM) strategies in regions where *B. tabaci* MEAM1 is a primary pest.

## Materials and Methods

### Whitefly Sampling and Establishment

**Fig. 1.**
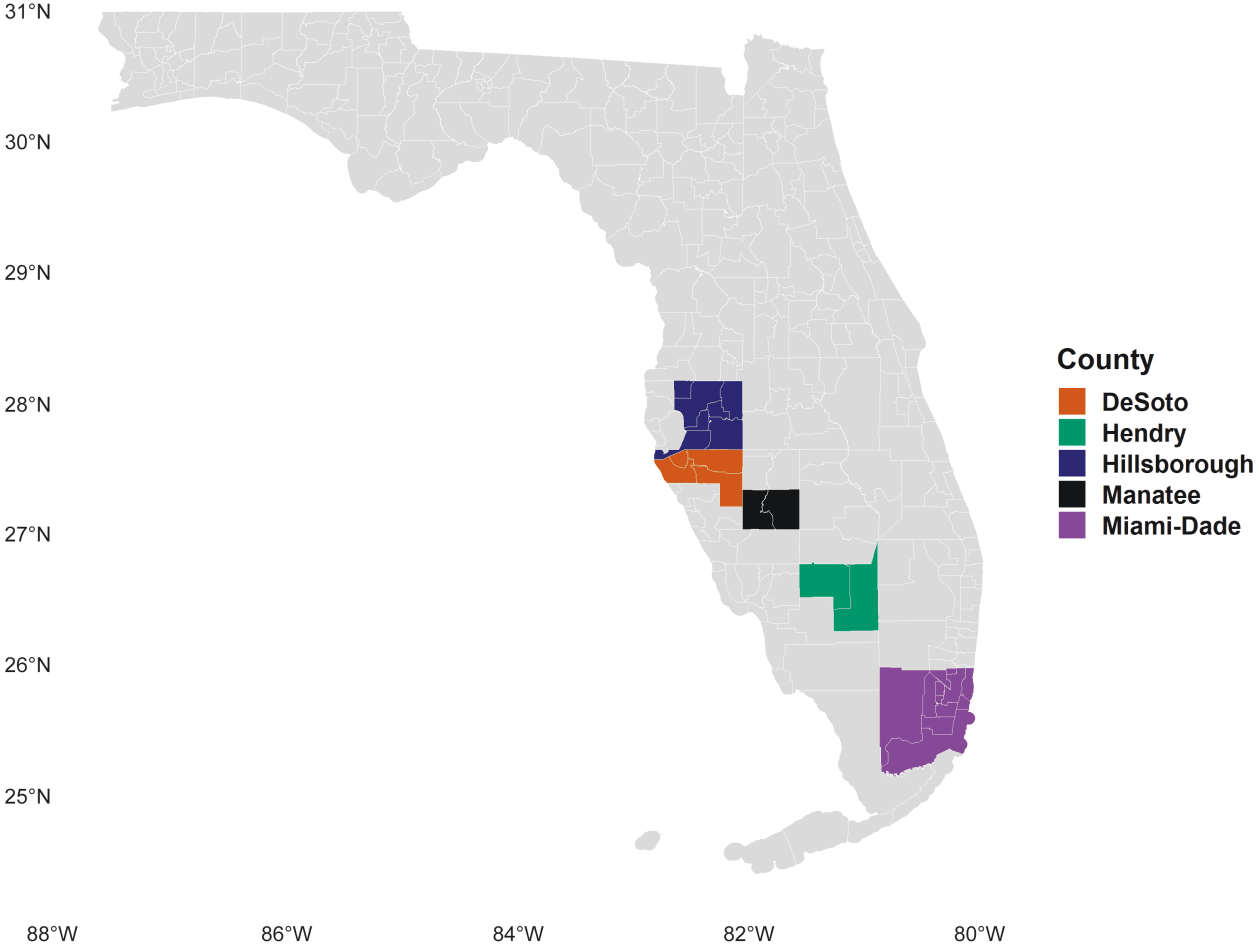
*Bemisia tabaci* MEAM1 populations collected from different counties in Florida.

Fifteen populations of *B. tabaci* (MEAM1) were collected predominantly from 5 Florida counties between Dec 2021 and Dec 2022, as illustrated in Fig. 1. These areas are categorized by heterogeneity in crop types, coupled with varying whitefly management strategies specific to different crops (Rossitto De Marchi et al. 2021).

Among these populations, 12 were collected from tomato, and one, each, was collected from Chinese okra (*Luffa acutangula* L.) (Cucurbitaceae), eggplant (*Solanum melongena* L.) (Solanaceae), and snap bean (*Phaseolus vulgaris* L.) (Fabaceae). Geographic coordinates and crop types of collection sites are summarized in Table 1. The susceptible reference colony has been maintained at the University of Florida (UF) Gulf Coast Research and Education Center (GCREC) in Wimauma, FL, USA, since the early 1990s, without exposure to insecticides.

**Table 1.** Field populations of *Bemisia tabaci* MEAM1 collected from commercial vegetable fields in south Florida and subjected to a serial dilution bioassay to test susceptibility of adults to afidopyropen, cyantraniliprole, dinotefuran, and flupyradifurone

Pop. ID	Collection date	Coordinates	Host crop
Manatee1	8-Dec-2021	27°35ʹ22ʹʹN 82°07ʹ15ʹʹW	Tomato
DeSoto1	8-Dec-2021	27°17ʹ18ʹʹN 82°02ʹ31ʹʹW	Tomato
Hendry1	9-Feb-2022	26°39ʹ27ʹʹN 81°25ʹ24ʹʹW	Tomato
Manatee2	15-Feb-2022	27°35ʹ14ʹʹN 82°07ʹ06ʹʹW	Tomato
Hillsborough1	15-Feb-2022	27°45ʹ27ʹʹN 82°23ʹ46ʹʹW	Tomato
Hendry2	16-Feb-2022	26°39ʹ32ʹʹN 81°22ʹ26ʹʹW	Tomato
DeSoto2	16-Feb-2022	27°16ʹ16ʹʹN 82°0ʹ24ʹʹW	Tomato
DeSoto3	16-Feb-2022	27°17ʹ41ʹʹN 82°02ʹ33ʹʹW	Tomato
MiamiDade1	19-Dec-2022	25º36ʹ34ʹʹN 80º28ʹ46ʹʹW	Snap beans
MiamiDade2	19-Dec-2022	25º36ʹ36ʹʹN 80º28ʹ47ʹʹW	Eggplant
MiamiDade3	20-Dec-2022	25º29ʹ28ʹʹN 80º31ʹ49ʹʹW	Tomato
MiamiDade4	20-Dec-2022	25º29ʹ56ʹʹN 80º30ʹ44ʹʹW	Tomato
MiamiDade5	20-Dec-2022	25º32ʹ19ʹʹN 80º31ʹ38ʹʹW	Tomato
MiamiDade6	20-Dec-2022	25º35ʹ47ʹʹN 80º27ʹ52ʹʹW	Chinese okra

^a^Number of *B. tabaci* adults exposed to various concentrations.

^b^Lethal concentration of the active ingredient required to kill 50% of tested individuals.

Whitefly adults were aspirated from crops on commercial vegetable farms into 2 ml glass eyedroppers (Fisher Scientific, Waltham, MA, USA). To facilitate collection and confine the whiteflies, a sponge was placed at the wide end of the eyedropper. Subsequently, after gathering 10 whitefly adults, each eyedropper was sealed with Parafilm (Sigma, Saint Louis, MO, USA). Over 100 whiteflies were aspirated from each site to establish a colony. Cages (24.5 × 24.5 × 63.0 cm, BugDorm-4M2260, MegaView Science Co., Ltd, Taichung, Taiwan) containing Delta Pine 143 B2RF cotton plants with 6 to 8 true leaves were brought to the collection sites to host the whiteflies. To release the collected whiteflies onto cotton plants, the sponges were removed from the eyedroppers, which were then positioned at the base of the potted cotton plants. Following each collection, the cages containing the whitefly adults were transported to the UF GCREC in Wimauma, FL, USA. These cages were maintained in growth rooms at 26.6 °C (± 1.2 °C), 40%–50% relative humidity, and a 16:8 (light:dark) photoperiod. Under these conditions, whitefly populations required 6 to 7 wk to establish on cotton plants and produce sufficient adult offspring for testing.

### Species Identification

Twenty individual whiteflies from each field-collected population were separated to extract the total nucleic acids following a modified Chelex protocol (Walsh et al. 2013). Whitefly adults were homogenized in a 1.5 ml-tube containing 40 μl of a 5% Chelex solution. The tube was vortexed for 10 s, followed by incubation at 56 °C for 15 min and 99 °C for 8 min. Subsequently, after centrifugation at 13,000 rpm for 5 min, the supernatant was collected and utilized as a template for PCR amplification. In the PCR analysis, the primer pair Bem23F (5ʹ-CGGAGCTTGCGCCTTAGTC-3ʹ) and Bem23R (5ʹ-CGGCTTTATCATAGCTCGT-3ʹ) (Thermo Fisher Scientific, Waltham, MA, USA) were employed to distinguish between MEAM1 and MED whitefly species. These primers amplify a microsatellite locus, yielding approximately 200 and 400 bp products for MEAM1 and MED, respectively (De Barro et al. 2002, Kontsedalov et al. 2012). The amplification process involved an initial phase at 95 °C for 3 min, followed by 35 cycles of 95 °C for 1 min, annealing at 55 °C for 1 min, and extension at 72 °C for 1 min, concluding with a final extension phase at 72 °C for 5 min. Visualization of the PCR products was achieved through electrophoresis in a 2% agarose gel stained with Gel Red.

### Adult Serial Dilution Bioassay

Bioassays were conducted using cotton plants as host for whitefly adults as recommended by the IRAC Method 015 v4 (https://irac-online.org/methods/trialeurodes-vaporariorum-bemisia-tabaci-adult/?ext=pdf). Cotton plants were cultivated in insect-free growth rooms under controlled conditions (26.6 °C ± 1.2 °C, 40%–50% RH, and a 16:8 light:dark cycle). Two plants, each, were cultivated in 10-cm-diameter pots filled with Fafard Professional Potting Mix soil (Agawam, MA, USA). These plants received a single application of 45 g of Osmocote Plus 15-9-12 fertilizer (The Scotts Company, Marysville, OH, USA) and were watered 3 times weekly. For the bioassays, cotton plants with 6 to 8 true leaves were chosen.

Insecticide solutions, each containing 500 ml of deionized water and varying concentrations of dinotefuran (Venom, Valent USA, Walnut Creek, CA, USA), flupyradifurone (Sivanto Prime, Bayer Corporation, St. Louis, MO, USA), afidopyropen (Sefina, BASF, Research Triangle Park, NC, USA), or cyantraniliprole (Exirel, FMC Corporation, Philadelphia, PA, USA), were prepared in beakers. The tested concentrations varied for each whitefly population. In the initial phase, 4 concentrations ranging from 0.01 to 500 mg l^−1^ were assessed through serial dilution to establish lower and upper reference concentrations for each population. Subsequently, a second series of 4 concentrations was evaluated based on the outcomes of the initial test to establish concentration–response curves. In total, we utilized 5 to 7 concentrations to generate concentration–response data to estimate the concentration required to obtain 50% population mortality (LC_50_). We calculated and pipetted the concentration needed to achieve the desired amount of each insecticide concentration in mg l^−1^ (ppm). Cotton leaves were taken from the growth room and cut into discs to fit snugly into 150 × 15 mm Petri dishes (Fisher Scientific item # FBO875714, Waltham, MA, USA). Then, each cotton leaf disc was individually immersed in its respective insecticide solution for approximately 20 s. These leaf discs were then left to air dry for about 2 h, then directly positioned in a Petri dish for the infestation process.

Adult whiteflies from the tested populations were aspirated in groups of 10 into glass eyedroppers from the cotton plants. They were chilled in the freezer for 60 s to reduce movement and then gently tapped into each Petri dish. A single Petri dish, containing a treated cotton leaf and 10 adult whiteflies, served as an experimental unit. Every concentration was replicated 4 or 5 times. After 72 h of treatment, the Petri dishes were examined under a stereomicroscope, and the number of live and dead whitefly adults was recorded.

### Statistical Analysis

The LC_50_ of *B. tabaci* adults was determined using probit analysis with Polo Suite software v.4 (LeOra Software, Berkshire, UK). The software requires 3 inputs for each concentration tested: the specified insecticide concentration in parts per million (ppm) or mg l^−1^; the total number of insects counted (live + dead); and the number of whitefly adults that responded to the tested concentration (dead). Natural response was used as a parameter, and doses were logarithmically scaled using Polo when certain populations exhibited heterogeneity factors >1.0 (Robertson et al. 2017). Trials with control mortality exceeding 20% were excluded and repeated (Robertson et al. 2017).

Resistance ratios (RR) were calculated by dividing the LC_50_ values of each population by the LC_50_ value of the susceptible Lab MEAM1 population (Robertson et al. 2017). For discussion purposes and to establish a conservative approach, we created a rationale to attribute a terminology for “resistance” and “susceptibility”. Populations with RR values below 15 were categorized as susceptible. Populations with RR values falling within 15 to 45 were classified as low resistant. Populations with RR values ranging from 45 to 90 were considered moderately resistant. Populations with RR values above 90 were categorized as highly resistant.

## Results

### Species Identification

The PCR product visualized on a 2% agarose gel exhibited a microsatellite locus amplification of ~200 bp for all specimens collected from each population. Thus, all populations were identified as *B. tabaci* MEAM1. These whitefly populations originated from various counties across south Florida. Most populations were collected from tomato, while MiamiDade1 (snap beans), MiamiDade2 (eggplant), and MiamiDade6 (Chinese okra) were collected from other vegetable crops (Table 1).

### Resistance Profile to Dinotefuran

The bioassays conducted to study resistance to dinotefuran showed that 3 days after treatment, the percentage of live whitefly adults in the susceptible lab colony (Lab MEAM1) used as control ranged from 87% to 97.3%. A baseline LC_50_ value of 1.74 mg a.i. l^−1^ (95% CI: 1.10–3.05) was established.

Populations were sorted based on ascending RRs, as presented in Table 2. All populations tested, including MiamiDade2 (RR: 1), MiamiDade6 (RR: 1), MiamiDade3 (RR: 1), Manatee1 (RR: 2), MiamiDade4 (RR: 2), MiamiDade5 (RR: 2), Hendry2 (RR: 2), DeSoto1 (RR: 3), MiamiDade1 (RR: 3), Hendry1 (RR: 6), DeSoto2 (RR: 6), DeSoto3 (RR: 6), Manatee2 (RR: 8), and Hillsborough1 (RR: 8), demonstrated susceptibility to dinotefuran, as indicated by their RRs < 15 (Table 2).

**Table 2.** LC_50_ values in different populations of *Bemisia tabaci* MEAM1 for the insecticidal dinotefuran

Population	*N* ^a^	Slope (± SE)	LC_50_ (mg a.i. l^−1^) (95% FL)^b^	*X* ^2^	DF	Resistance ratio^c^
Lab MEAM1	275	1.12 (± 0.21)	1.74 (1.10–3.05)	0.43	2	—
MiamiDade2	301	0.94 (± 0.15)	1.81 (0.88–3.59)	1.62	3	1
MiamiDade6	283	0.53 (± 0.09)	1.90 (1.09–2.67)	1.05	2	1
MiamiDade3	260	0.98 (± 0.18)	1.93 (0.77–3.24)	1.42	2	1
Manatee1	339	1.46 (± 0.22)	2.95 (2.49–3.41)	0.35	4	2
MiamiDade4	397	0.25 (± 0.04)	3.02 (1.76–4.12)	0.55	2	2
MiamiDade5	451	4.35 (± 0.77)	3.57 (3.37–3.76)	0.12	2	2
Hendry2	349	1.94 (± 0.34)	4.25 (2.92–5.65)	1.34	3	2
DeSoto1	341	2.16 (± 0.33)	4.61 (3.18–6.19)	0.54	2	3
MiamiDade1	379	1.38 (± 0.20)	4.98 (2.49–8.34)	2.29	3	3
Hendry1	370	0.09 (± 0.02)	10.45 (8.59–11.93)	0.16	2	6
DeSoto2	344	1.42 (± 0.25)	10.55 (7.92–14.79)	0.80	3	6
DeSoto3	378	0.05 (± 0.01)	11.05 (9.56–12.32)	0.05	2	6
Manatee2	268	1.83 (± 0.41)	13.08 (9.97–16.20)	0.19	2	8
Hillsborough1	302	1.47 (± 0.37)	13.81 (12.31–15.43)	0.04	2	8

^a^Number of *B. tabaci* adults exposed to various concentrations.

^b^Lethal concentration of the active ingredient required to kill 50% of tested individuals.

^c^LC_50_ relative to the susceptible (laboratory) population.

### Resistance Profile to Flupyradifurone

In the bioassays examining flupyradifurone resistance, the percentage of live whitefly adults in the susceptible lab colony used as control ranged from 84% to 97.9% 3 days posttreatment. A baseline LC_50_ value of 3.07 mg a.i. l^−1^ (95% CI: 2.66–3.56) for flupyradifurone was established for the reference population, Lab MEAM1. Populations were organized in ascending order of RRs, as outlined in Table 3. All field populations, including MiamiDade4 (RR: 2), MiamiDade6 (RR: 2), MiamiDade3 (RR: 3), MiamiDade2 (RR: 3), MiamiDade1 (RR: 4), Hendry2 (RR: 5), Hendry1 (RR: 5), DeSoto2 (RR: 5), DeSoto1 (RR: 5), Manatee1 (RR: 6), MiamiDade5 (RR: 7), DeSoto3 (RR: 8), and Hillsborough1 (RR: 8.), exhibited susceptibility to flupyradifurone (Table 3).

**Table 3.** LC_50_ values in different populations of *Bemisia tabaci* MEAM1 for the insecticidal flupyradifurone

Population	*N* ^a^	Slope (± SE)	LC_50_ (mg a.i. l^−1^) (95% FL)^b^	*X* ^2^	DF	Resistance ratio^c^
Lab MEAM1	386	1.12 (± 0.21)	3.07 (2.66–3.56)	0.12	3	—
MiamiDade4	340	0.15 (± 0.02)	5.30 (4.22–6.30)	0.15	2	2
MiamiDade6	280	1.55 (± 0.31)	5.67 (4.67–6.73)	0.12	2	2
MiamiDade3	326	0.11 (± 0.02)	8.58 (5.95–11.39)	1.46	3	3
MiamiDade2	363	1.40 (± 0.23)	9.32 (7.35–11.48)	0.38	3	3
MiamiDade1	356	1.02 (± 0.24)	12.14 (9.53–15.45)	0.26	3	4
Hendry2	268	0.05 (± 0.01)	14.12 (10.15–21.58)	0.31	2	5
Hendry1	300	1.47 (± 0.29)	14.17 (8.78–19.78)	0.85	3	5
DeSoto2	240	1.61 (± 0.36)	14.20 (11.98–16.71)	0.12	2	5
DeSoto1	349	1.05 (± 0.26)	15.27 (11.01–20.14)	0.32	3	5
Manatee1	349	4.12 (± 0.78)	17.16 (13.08–21.96)	2.84	3	6
MiamiDade5	381	0.04 (± 0.01)	22.76 (18.22–26.98)	0.73	3	7
DeSoto3	232	0.06 (± 0.01)	23.06 (18.93–26.84)	0.41	2	8
Hillsborough1	420	0.08 (± 0.01)	24.86 (20.75–28.31)	0.61	2	8

^a^Number of *B. tabaci* adults exposed to various concentrations.

^b^Lethal concentration of the active ingredient required to kill 50% of tested individuals.

^c^LC_50_ relative to the susceptible (laboratory) population.

### Resistance Profile to Afidopyropen

In the bioassays evaluating afidopyropen resistance, the percentage of live whitefly adults in the untreated control group ranged from 85.7% to 97% 3 days after treatment. The reference population, Lab MEAM1, set a baseline with an LC_50_ value of 0.52 mg a.i. l^−1^ (95% CI: 0.38–0.69), highlighting its high susceptibility to afidopyropen. Populations were organized by ascending RRs, as detailed in Table 4. The populations MiamiDade6 (RR: 1), DeSoto1 (RR: 2), MiamiDade4 (RR: 3), MiamiDade1 (RR: 4), Hendry2 (RR: 4), Manatee1 (RR: 5), DeSoto3 (RR: 6), MiamiDade2 (RR: 6), MiamiDade5 (RR: 7), and Hendry1 (RR = 9), displayed a high susceptibility to afidopyropen. In contrast, the populations Hillsborough1 (RR: 38) and DeSoto2 (RR: 51), each collected from tomato, exhibited low and moderate resistance, respectively (Table 4).

**Table 4.** LC_50_ values in different populations of *Bemisia tabaci* MEAM1 for the insecticidal afidopyropen

Population	*N* ^a^	Slope (± SE)	LC_50_ (mg a.i. l^−1^) (95% FL)^b^	*X* ^2^	DF	Resistance ratio^c^
Lab MEAM1	269	0.52 (± 0.17)	0.52 (0.38–0.69)	0.04	2	—
MiamiDade6	271	1.17 (± 0.23)	0.57 (0.32–0.91)	0.36	2	1
DeSoto1	328	1.70 (± 0.24)	1.0 (0.70–1.33)	1.02	3	2
MiamiDade4	369	1.19 (± 0.35)	1.62 (0.97–2.30)	0.26	2	3
MiamiDade1	357	1.35 (± 0.24)	2.01 (1.80–2.24)	0.10	3	4
Hendry2	289	1.49 (± 0.31)	2.02 (1.16–2.81)	0.41	2	4
Manatee1	438	1.15 (± 0.18)	2.74 (1.60–4.54)	1.85	3	5
DeSoto3	288	0.77 (± 0.22)	2.91 (1.10–5.32)	0.43	2	6
MiamiDade2	388	1.26 (± 0.21)	3.20 (1.46–5.43)	2.43	3	6
MiamiDade5	481	0.93 (± 0.15)	3.48 (2.81–4.35)	0.44	4	7
Hendry1	534	0.99 (± 0.19)	4.71 (4.52–4.88)	0.04	2	9
Hillsborough1	250	1.69 (± 0.43)	19.64 (14.76–26.28)	0.20	2	38
DeSoto2	299	1.98 (± 0.48)	26.68 (21.80–33.56)	0.27	2	51

^a^Number of *B. tabaci* adults exposed to various concentrations.

^b^Lethal concentration of the active ingredient required to kill 50% of tested individuals.

^c^LC_50_ relative to the susceptible (laboratory) population.

### Resistance Profile to Cyantraniliprole

In the bioassays assessing cyantraniliprole resistance, the untreated control treatment revealed 87% to 99% live whitefly adults 3 days after treatment. The reference population, Lab MEAM1, established a baseline with an LC_50_ value of 0.60 mg a.i. l^−1^ (95% CI: 0.31–0.88). Populations were ordered based on increasing RRs, as outlined in Table 5. Populations of *B. tabaci* MEAM1 exhibited varying susceptibility to cyantraniliprole, as detailed in Table 5. Among the populations, MiamiDade6 (RR: 3), DeSoto1 (RR: 9), MiamiDade2 (RR: 9.5), MiamiDade4 (RR: 10), MiamiDade3 (RR: 10), and MiamiDade1 (RR: 10) displayed RR < 15, indicating susceptibility to cyantraniliprole. On the other hand, populations Hendry1 (RR: 21), DeSoto3 (RR: 28), and Manatee2 (RR: 29). all collected from tomato fields, exhibited low resistance to cyantraniliprole. Furthermore, populations MiamiDade5 (RR: on), Hendry2 (RR: 52), Manatee1 (RR: 57), DeSoto2 (RR: 64), and Hillsborough1 (RR: 78), all derived from tomato fields, demonstrated moderate resistance to cyantraniliprole.

**Table 5.** LC_50_ values in different populations of *Bemisia tabaci* MEAM1 for the insecticidal cyantraniliprole

Population	*N* ^a^	Slope (± SE)	LC_50_ (mg a.i. l^−1^) (95% FL)^b^	*X* ^2^	DF	Resistance ratio^c^
Lab MEAM1	347	2.09 (± 0.35)	0.60 (0.31–0.88)	1.33	2	—
MiamiDade6	293	1.05 (± 0.22)	1.97 (1.20–2.92)	0.31	2	3
DeSoto1	551	2.06 (± 0.27)	5.55 (3.80–7.12)	2.37	4	9
MiamiDade2	431	0.83 (± 0.13)	5.70 (4.90–6.58)	0.15	4	9.5
MiamiDade4	290	1.60 (± 0.30)	5.81 (4.05–7.51)	0.24	2	10
MiamiDade3	382	1.80 (± 0.35)	5.82 (4.19–7.48)	0.32	2	10
MiamiDade1	320	1.03 (± 0.23)	6.09 (3.90–8.94)	0.29	2	10
Hendry1	275	1.45 (± 0.24)	12.72 (10.91–14.57)	0.15	3	21
DeSoto3	256	0.04 (± 0.01)	16.77 (10.02–23.20)	0.69	2	28
Manatee2	259	1.38 (± 0.38)	17.64 (16.06–19.53)	0.02	2	29
MiamiDade5	439	1.30 (± 0.22)	28.13 (23.25–33.94)	0.35	3	47
Hendry2	280	2.35 (± 0.64)	31.40 (23.98–45.39)	0.10	2	52
Manatee1	281	0.03 (± 0.01)	34.16 (27.57–35.93)	0.47	2	57
DeSoto2	269	0.03 (± 0.01)	38.24 (32.56–45.49)	0.27	2	64
Hillsborough1	310	0.03 (± 0.01)	46.59 (42.80–50.70)	0.11	2	78

^a^Number of *B. tabaci* adults exposed to various concentrations.

^b^Lethal concentration of the active ingredient required to kill 50% of tested individuals.

^c^LC_50_ relative to the susceptible (laboratory) population.

## Discussion

The emergence of resistance in insect populations poses a significant challenge to integrated pest management (IPM). In this study, we investigated the susceptibility of *B. tabaci* MEAM1 populations to 4 key insecticides used to manage this pest in Florida: afidopyropen, cyantraniliprole, dinotefuran, and flupyradifurone. These active ingredients are routinely used to manage *B. tabaci* in Florida (Caballero et al. 2013, Smith and Giurcanu 2013, 2014, Smith and Nagle 2014, Smith et al. 2016, Sparks et al. 2020, Rossitto De Marchi et al. 2021). In our study, 14 whitefly populations from commercial vegetable fields, primarily tomato, were tested via serial dilution bioassays. This dataset encompassed both LC_50_ and RR to document resistance levels for all tested populations. Our data indicated that some populations exhibited varying degrees of resistance to different active ingredients. Practical resistance can be defined as resistance developed in the field that diminishes the effectiveness of a pesticide, leading to consequences for pest management such as reduced efficacy of a pesticide (Tabashnik et al. 2014). Based on trends observed in our data, coupled with the resistance terminology defined by Tabashnik et al. (2014), we determined the following terminology in the context of our study: “low resistance” indicates early signs of reduced sensitivity of a population to a pesticide; moderate resistance reflects an intermediate resistance level of a population to a pesticide, which involves initial signs of reduced control efficacy; and high resistance signifies robust and firmly established resistance, which includes significant reduction in control efficacy but lacks evidence of field control failure.

Our findings revealed that dinotefuran remains an effective option for managing *B. tabaci* MEAM1 populations. All field-collected populations have displayed susceptibility to dinotefuran, with RRs ranging from 1 to 8. This consistent efficacy suggests that dinotefuran continues to offer robust control over MEAM1 populations in these south Florida regions. Since its commercial introduction in 2005, dinotefuran has been consistently efficient in managing *B. tabaci* MEAM1 across different regions of the United States, including Florida, Georgia, California, and Arizona (Prabhaker et al. 2005, Smith and Nagle 2014, Smith et al. 2016, Sparks et al. 2020, Rossitto De Marchi et al. 2021, Cremonez et al. 2023). Moreover, the continuous efficacy of dinotefuran against *B. tabaci* MEAM1 has been reported globally (Qu et al. 2017, Hopkinson and Pumpa 2019, Barakat et al. 2022). This is particularly encouraging given the economic importance of vegetable crops that are vulnerable to *B. tabaci* MEAM1 damage, especially in south Florida. The inclusion of dinotefuran within the rotation scheme for modes of action, as recommended by IRAC, remains one of the most effective alternatives for whitefly control in Florida.

As a nAChR competitive modulator, dinotefuran effectively stimulates nAChRs, leading to an involuntary excitatory response in the nervous system of insects and eventually killing them. Dinotefuran contains a tetrahydrofuran ring, which is believed to impart unique properties and action mechanisms to the compound (Wakita 2008). According to Wakita (2008), this structural distinction as well as its high water solubility allow for a differentiation in its mode of action compared to other neonicotinoids. Additionally, *B. tabaci* MEAM1 does not seem to detoxify dinotefuran via the microsomal monooxygenases, including the cytochrome P450 monooxygenase (P450) gene CYP6CM1, which has been implicated in resistance to other neonicotinoids such as imidacloprid (Hamada et al. 2019). These features combined with its systemic activity make dinotefuran especially potent against a broad spectrum of hemipteran insects that feed on plant sap. While dinotefuran remains effective, its judicious use is essential. For effective pest management and to preserve its long-term viability, dinotefuran applications in crops are generally administered at planting, through drip irrigation, or as a foliar treatment (Smith et al. 2018). Continuous monitoring of *B. tabaci* MEAM1 susceptibility to dinotefuran is critical for early detection of resistance development and for timely evaluations of IPM strategies.

Similarly to dinotefuran, flupyradifurone demonstrated effectiveness against *B. tabaci* MEAM1 adults in our study. Resitance ratios ranged from 2 to 8, indicating that all field-collected populations remain susceptible to flupyradifurone. The low RRs and its continued efficacy suggest that flupyradifurone remains a valuable tool for *B. tabaci* MEAM1 management in south Florida. Over the past decade, flupyradifurone has shown an overall high efficacy in controlling *B. tabaci* MEAM1 and TYLCV in Florida, Georgia, and Arizona (Smith and Giurcanu 2013, 2014, Smith and Nagle 2014, Castle et al. 2017, Sparks et al. 2020, Rossitto De Marchi et al. 2021, Cremonez et al. 2023). Additionally, flupyradifurone efficacy against *B. tabaci* MEAM1 and MED has been recently reported in other countries (Roditakis et al. 2017, Guo et al. 2020, Maluta et al. 2020, Liu et al. 2021, Issa et al. 2022). Nonetheless, there is at least one report of varying degrees of resistance among Florida populations (Smith et al. 2016). Specifically, 5 populations from commercial tomato fields exhibited RRs for flupyradifurone that indicated moderate to high levels of resistance (55 to 147). Intriguingly, these findings occurred even though these populations were not previously exposed to flupyradifurone since this material became commercially available in 2015 (Smith et al. 2016). Nevertheless, the study by Smith et al. (2016) reported an LC_50_ of 0.010 (0.001–0.060) for the susceptible reference population, whereas the most resistant population from that study (Hendry1) exhibited an LC_50_ of 1.471 (0.802–2.477). Considering the RR criterion (147 in that case), Hendry1 population (Smith et al. 2016) can be classified as highly resistant to flupyradifurone.

A previous study demonstrated that flupyradifurone influenced the stylet activity of *B. tabaci* MEAM1, decreasing both the frequency and length of intracellular punctures and the consumption of phloem sap (Maluta et al. 2020). Nauen et al. (2014) attributed the distinctiveness of flupyradifurone to its butenolide pharmacophore, derived from the natural alkaloid stemofoline, differentiating it from other commercial nAChR agonists. Flupyradifurone does not undergo detoxification by the enzyme CYP6CM1, which is known to confer resistance to other neonicotinoids in whiteflies (Nauen et al. 2014). However, the flupyradifurone resistance report by Smith et al. (2016) suggested that this material could potentially exhibit cross-resistance with other neonicotinoid insecticides. Considering its demonstrated efficacy, flupyradifurone must be used judiciously by incorporating this material within a rotation of different modes of action to mitigate potential resistance development. Due to similarity with the mode of action of neonicotinoids (IRAC group 4A), flupyradifurone should be included in the same group when considering rotating modes of action for IRM strategies (Sparks and Nauen 2015, Smith et al. 2018).

Afidopyropen displays translaminar activity with apparent limited systemic effects (Jeschke 2021, Horikoshi et al. 2022). Afidopyropen is a novel chordotonal modulator that interferes with insect feeding and movement by hyperactivation and subsequent silencing of transient receptor potential vanilloid (TRPV) channels (Horikoshi et al. 2022). This silencing of TRPV channels affects the function of chordotonal organs, leading to a loss of coordination in insects, which indirectly impairs their ability to feed, resulting in desiccation and death (Horikoshi et al. 2022). Detoxifying P450 enzymes and overexpression of the TRPV Nan gene might play a role in the development of afidopyropen resistance in *B. tabaci* (Wang, Zhang, et al. 2022, Wang et al. 2023). Given the observed variability in response to afidopyropen in our study and its unique mode of action, there is an evident need for targeted monitoring. Adapting IRM strategies is crucial to ensure that afidopyropen remains an effective and viable tool for controlling *B. tabaci* MEAM1 in south Florida and elsewhere.

In our study, afidopyropen exhibited a distinct resistance profile among the tested *B. tabaci* MEAM1 populations. While over 80% of the populations showed susceptibility, 2 populations displayed low (RR: 38) and moderate (RR: 51) resistance, respectively. The low and moderate afidopyropen-resistant populations collected from Hillsborough and DeSoto counties, respectively, have also demonstrated moderate degrees of resistance to cyantraniliprole, which may indicate potential cross-resistance. Intriguingly, populations from these 2 counties have also demonstrated moderate to extremely high levels of resistance to spirotetramat (Dimase et al. 2024). For these reasons, pest management practices must be urgently reviewed in these areas, and further studies are warranted to investigate potential cross-resistance between afidopyropen and cyantraniliprole among *B. tabaci* MEAM1 populations in Florida. This is the first documentation of baseline suceptibility data of *B. tabaci* MEAM1 populations from vegetable fields to afidopyropen in the United States and the first report of initial levels (low to moderate) of resistance in the country. Resistance development to afidopyropen in south Florida suggests that certain *B. tabaci* MEAM1 populations may be under increased selection pressure from this insecticide. Initial studies from China demonstrated that afidopyropen was highly effective against both *B. tabaci* MEAM1 and MED populations (Zhang et al. 2021, Zhou et al. 2021). However, a more recent study reported that a *B. tabaci* MED population from Haidan, a district of Beijing in China, exhibited moderate resistance to afidopyropen, little cross-resistance to cyantraniliprole, and a moderate degree of cross-resistance to sulfoxaflor (Wang, Gao, et al. 2022). Furthermore, 4 *B. tabaci* Asia I populations from India (~23.5%) displayed moderate resistance to afidopyroene; however, cross-resistance to cyantraniliprole was not observed (Mahalanobish et al. 2022).

In the current study, cyantraniliprole displayed a diverse range of efficacy across the tested populations. While 57% of populations exhibited low to moderate resistance (RRs ranging from 21 to 78), the remaining populations were susceptible (RRs from 3 to 10). Interestingly, the populations exhibiting moderate resistance to cyantraniliprole were consistently identified across all 5 counties from which we collected whiteflies, and notably, all originated from tomato crops. While only one *B. tabaci* MEAM1 population collected from tomato in MiamiDade County was moderately resistant to cyantraniliprole, the remaining 4 populations from that county were consistently susceptible. This may indicate that crops and/or localized farming practices may contribute to the susceptibility status of *B. tabaci* MEAM1. Such variation in susceptibility to cyantraniliprole found in our study highlights the complexity of resistance dynamics within *B. tabaci* MEAM1 populations. A significant proportion of populations exhibiting low to moderate resistance to cyantraniliprole indicates emerging challenges for this insecticide in certain south Florida regions.

Earlier research in the United States highlighted cyantraniliprole’s unique effectiveness in controlling a range of pests including whiteflies, psyllids, leafminers, and aphids (Selby et al. 2013, Tiwari and Stelinski 2013). From 2008 to 2010, studies in Arizona, Florida, and Georgia reported that field-collected whiteflies exhibited complete susceptibility to cyantraniliprole and showed no cross-resistance to other insecticides (Li et al. 2012, Caballero et al. 2013, Riley and Srinivasan 2019). Moreover, cyantraniliprole has demonstrated its ability to reduce *B. tabaci* feeding, oviposition, and the transmission of TYLCV, making it an invaluable tool in pest management in the United States (Caballero et al. 2013, 2015, Cameron et al. 2014, 2016). Furthermore, a field study conducted in Florida has demonstrated cyantraliniprole’s capacity in boosting yields during seasons with reduced whitefly and virus pressure (Smith and Nagle 2014). Nevertheless, the efficacy of cyantraniliprole was found to be lower compared to dinotefuran and flupyradifurone, in a recent maximum dose bioassay study (Rossitto De Marchi et al. 2021). Internationally, the narrative follows a similar trend. Earlier studies from different locations (and *B. tabaci* species) such as China (MED) (Xie et al. 2014), Spain (MED) (Grávalos et al. 2015), and Australia (MEAM1) (Hopkinson and Pumpa 2019) reported a high susceptibility of *B. tabaci* populations to cyantraniliprole. However, recent reports have shown low to high resistance to this active ingredient in Brazil (MED/MEAM1) (Libardi Miraldo et al. 2021) and China (MED) (Zheng et al. 2017, Wang et al. 2018, Wang, Che, et al. 2022). The results from these studies highlight the importance of monitoring *B. tabaci* resistance to cyantraniliprole by crop and region.

Coupled with its systemic nature, cyantraniliprole allows applications both to the roots and foliage (Smith and Nagle 2014). When applied as foliar treatments, the effectiveness in reducing TYLCV infection was generally higher than when applied to the soil (Caballero et al. 2015). Laboratory trials on *B. tabaci* have shown that cyantraniliprole exhibits xylem-mobile activity (Caballero et al. 2013, Cameron et al. 2013). The mode of action of cyantraniliprole, which releases calcium from muscle cells, causes rapid feeding cessation leading to paralysis and eventual death in whitefly (Selby et al. 2013). This mechanism is believed to play a pivotal role in the reduced transmission of TYLCV (Civolani et al. 2014). Wang, Che, et al. (2022) documented that resistance to cyantraniliprole in some populations was associated with the overexpression of the CYP4G68 P450 gene. The correlation between calcium-binding proteins stabilizing Ca^2+^ concentration in whiteflies and cyantraniliprole exposure also plays a role into resistance mechanisms (Guo et al. 2019). Although resistance to cyantraniliprole was not previously documented in Florida’s whitefly populations, it has now emerged as a growing concern. Our study represents the first report of *B. tabaci* resistance to cyantraniliprole in the United States. Therefore, while cyantraniliprole is still an important tool in controlling *B. tabaci* populations, careful resistance management is crucial. Over-reliance or frequent cyantraniliprole applications could contribute to increased pressure for resistance development. A strategic IPM approach that employs cyantraniliprole judiciously, considering both its benefits and potential risks, will be essential in ensuring its long-term effectiveness. Cyantraniliprole should be applied during the later stages of crop growth to additionally target leafminers and caterpillars, while group 4 insecticides are best used at planting and in the immediate weeks that follow (Rossitto De Marchi et al. 2021). Monitoring the effectiveness of cyantraniliprole rigorously and adopting alternative pest management approaches are essential in areas experiencing emerging *B. tabaci* resistance.

Furthermore, the frequency of afidopyropen and cyantraniliprole use and, consequently, selection pressure will fluctuate among crops and local agricultural practices. The agricultural landscape of MiamiDade Co. is characterized by numerous crops, including several vegetables, tropical fruit groves, and ornamental nurseries that can host whiteflies throughout the entire year (Rossitto De Marchi et al. 2021). These fields are usually <30 ha and planted with different crops subjected to distinct whitefly management practices. This can result in distinct selection pressures in relation to locations with breaks in cropping cycles during the summer and winter months. For example, whiteflies found on weeds that surround fruit groves are not intensively sprayed, whereas whiteflies found in nurseries where vegetable and ornamental crops are cultivated are intensively sprayed. The agricultural scenario in MiamiDade Co. contrasts with larger-scale farms from Hillsborough-, DeSoto-, and Hendry Co., where insecticides are intensively used, and where we collected populations from. In summary, our study provides detailed insights into the resistance status of *B. tabaci* MEAM1 populations in south Florida to key insecticides. While dinotefuran and flupyradifurone maintain their effectiveness, the emergence of resistance to afidopyropen and cyantraniliprole in some populations highlights the importance of targeted monitoring and adaptive pest management strategies. Cross-resistance between certain insecticides may further complicate the resistance landscape. Sustainable pest management practices, including the exploration of alternative control strategies, are essential to reduce the reliance on chemical insecticides and ensure the long-term efficacy of pest management programs. These findings not only have implications for south Florida but also contribute to our understanding of *B. tabaci* resistance dynamics on a global scale. Collaborative efforts in pest management research are necessary to address the challenge of escalating resistance and to develop region-specific IRM strategies that safeguard the productivity of vegetable crops in areas where *B. tabaci* MEAM1 is a major pest. To ensure the continued utility of key insecticides, the agricultural community must remain proactive, well-informed, and adaptable to the evolving dynamics of *B. tabaci* resistance.
